# Retraction: Ph-triazole as a therapeutic agent for pancreatic cancer: Synthesis, in silico, and in vitro evaluation

**DOI:** 10.1371/journal.pone.0345609

**Published:** 2026-03-25

**Authors:** The PLOS One Editors

Following the publication of this article [1], concerns were raised that the NMR spectroscopy data pertaining to the characterization of Ph-triazole are not provided with [1], and that the Synthesis of the Ph-triazole subsection of the Materials and methods section provides insufficient detail regarding the “various spectroscopic techniques” used to purify and characterize Ph-triazole.

Upon editorial reassessment of [1], similarities were noted between multiple regions within multiple panels in Fig 9, and it was noted that the individual-level data underlying the graphs presented in Figs 1, 3-4, 7-10, and S1-S3 were not provided with [1].

The corresponding author’s response did not resolve the journal’s concerns.

In light of the above concerns, which call into question the integrity and reliability of the published results, the *PLOS One* Editors retract this article.

LZ responded but expressed neither agreement nor disagreement with the editorial decision. NZ, XL, FL, JJ, and TZ either did not respond directly or could not be reached.
